# Dual energy x-ray absorptiometry analysis contributes to the prediction of hip osteoarthritis progression

**DOI:** 10.1186/ar2845

**Published:** 2009-11-02

**Authors:** Martha C Castaño Betancourt, Jacqueline C Van der Linden, Fernando Rivadeneira, Rianne M Rozendaal, Sita M Bierma Zeinstra, Harrie Weinans, Jan H Waarsing

**Affiliations:** 1 Orthopaedic Research Laboratory, Erasmus Medical Center, Dr Mollewaterplein 50, 3000 CA, Rotterdam. The Netherlands; 2 Department of Internal Medicine, Erasmus Medical Center, Dr Mollewaterplein 50, 3000 CA, Rotterdam, The Netherlands; 3 Department of General Practice, Erasmus Medical Center, Dr. Mollewaterplein 50, 3000 CA, Rotterdam, The Netherlands; 4 Department of Epidemiology, Erasmus Medical Center, Dr. Mollewaterplein 50, 3000 CA, Rotterdam, The Netherlands

## Abstract

**Introduction:**

To determine if structural bone parameters obtained from dual energy X-ray absorptiometry (DXA) contribute to the prediction of progression of hip osteoarthritis (OA) and to test if the difference between the most affected (OA) hip and the contralateral hip adds to this prediction.

**Methods:**

The study group involves a prospective cohort of 189 patients that met the American College of Rheumatology (ARC) classification criteria for hip osteoarthritis. Progression was defined as 20% joint space narrowing or total hip replacement within a two years follow up. Software was developed to calculate geometrical aspects and bone mineral density (BMD) in different regions of interest of the proximal femur. Logistic regression was used to test if Kellgren and Lawrence (K-L) scores and DXA parameters can predict *progression *of OA. Models were compared using -2log likelihood tests, R^2 ^Nagelkerke and areas under the Receiver Operator Characteristic curves, assessed using 10-fold cross validation.

**Results:**

The model that included the DXA variables was significantly better in predicting hip OA progression than the model with K-L score of the affected side alone (*P *< 0.01). The addition of the differences in DXA parameters between the most affected and contralateral hip in the superior part of the femoral head, trochanteric and intertrochanteric area further improved the prediction of progression (*P *< 0.05). K-L score of the affected side was still the most significant single variable in the models.

**Conclusions:**

DXA parameters can significantly contribute to the prediction of progression in patients with hip osteoarthritis. The analysis of the DXA differences between the hips of the patient represents a small but significant contribution to this prediction. These analyses show the importance of bone density changes in the etiology of OA.

## Introduction

Osteoarthritis (OA) is a degenerative joint disease characterized by progressive damage of the articulate cartilage, occasional inflammation of the synovium, osteophytosis and alterations in the subchondral bone. It is often hypothesized that subchondral bone changes play an important role in either initiation or progression of osteoarthritis [1,2]. Changes in bone shape, bone mineral density (BMD) and subchondral bone mechanical properties were reported in the presence of radiographic signs of hip OA [3-8]. A number of studies were performed that correlate radiographic osteoarthritis and/or clinical symptoms with bone measurements based on dual energy X-ray absorptiometry (DXA) that are typically performed in relation to osteoporosis. These measures concern BMD in the hip or spine at specific regions of interest such as e.g. the femoral neck. This data is rather confusing and conflicting in many aspects. An increased local and remote BMD has been reported in patients with radiographic hip OA [9], suggesting an inverse relationship between osteoarthritis and osteoporosis. This was confirmed by Goker et al. [10] in patients that underwent total hip replacement, where the subjects with high progression of Joint Space Narrowing (JSN) at their contralateral hip had elevated BMD in both hip and spine.

Antoniades et al. only found this inverse relationship between local BMD and osteophytosis and not with JSN [11]. Other studies report an inverse relationship only in the affected hip and even a decreased BMD at remote sites and the contralateral hip [12,13]. This was further substantiated by Sandini et al, finding higher bone mineral content (BMC) and larger area in the DXA data from patients with hip OA [14]. Changed muscle conditions and weight bearing may alter the load conditions in OA and local bone density changes may be the result of adaptation to an altered load distribution through the bone structure. Altogether, there seems to be conflicting data concerning the relationship between bone related parameters in OA. The variables that have been analyzed using DXA are often defined only in regions of interest such as the femoral neck and vertebral body that are relevant for osteoporosis, for which DXA has been specifically designed. Beck and coworkers have designed methods to analyze a number of other parameters that are related to biomechanical aspects of the narrowest region of the proximal femur, an area of high interest in osteoporosis [15]. However, for OA other regions might be of more interest, such as the subchondral bone BMC or BMD.

The rate of progression of hip OA varies largely between patients. Some patients with radiographic signs of initial hip OA do not show disease progression for years. In other cases the disease progresses relatively fast, e.g. needing total hip replacement after less than two years after onset of the first symptoms. The determinants of this progression are largely unknown [16]. It is also unclear what the role is of BMD, BMC or morphological bone variations on progression of hip OA. Better understanding of the involvement of alterations in the bone might allow early identification of cases and maybe even provide opportunities for early intervention. Therefore, this study aims to determine if structural bone geometry and density parameters as determined by hip DXA scans in the proximal femur, contribute to the prediction of OA progression. Furthermore, we tested if the difference in these DXA-based variables between the most affected and contralateral hip adds to this prediction. Since left-right differences are independent of biological variation in bone size or density we hypothesize that these are better predictors of disease progression.

## Materials and methods

### Study population

This study includes primary care patients with osteoarthritis of the hip derived from the *glucosamine sulphate in hip osteoarthritis *(GOAL) trial of the Erasmus Medical Center, with data collected at baseline and every three months up to two years follow-up. Details of the study have been described earlier [17]. In summary, patients were eligible for inclusion in the GOAL cohort when they met one of the American College of Rheumatology (ACR) criteria for hip OA [18]. Patients that had already undergone hip replacement surgery or those on the waiting list for joint replacement were not included in the study. In addition, eligible patients with a Kellgren & Lawrence (K-L) score of 4, people with renal and/or hepatic disease, diabetes mellitus or with disabling co-morbidity were excluded. Sex, age, height, weight, duration of complaints and body mass index (BMI) were registered or measured in all OA patients. For this study only participants with bilateral radiographs and dual energy X-ray absorptiometry (DXA scans) of adequate quality measured at baseline and after two years follow up were included in the analyses. The Ethical Committee of Erasmus MC approved the study protocol, and patients provided written informed consent.

### Radiographic assessments

A strict protocol was used to enable correct measurements of joint space narrowing at baseline and two years follow up. Pelvic radiographs were taken in weight bearing position with the patient's hips at 15° internal rotation. From the digitized x-rays the minimal joint space width (JSW) was assessed at the medial, axial, superior and lateral points of the joint or any other site where the JSW was minimal. The intraclass correlation coefficient of the minimal joint space width measurement was 0.98. All the radiographs were scored at baseline according to the Kellgren-Lawrence score from grades from 0 *no osteoarthritis *to 4 *severe osteoarthritis *[19].

### DXA scan analysis

**Figure 1:**
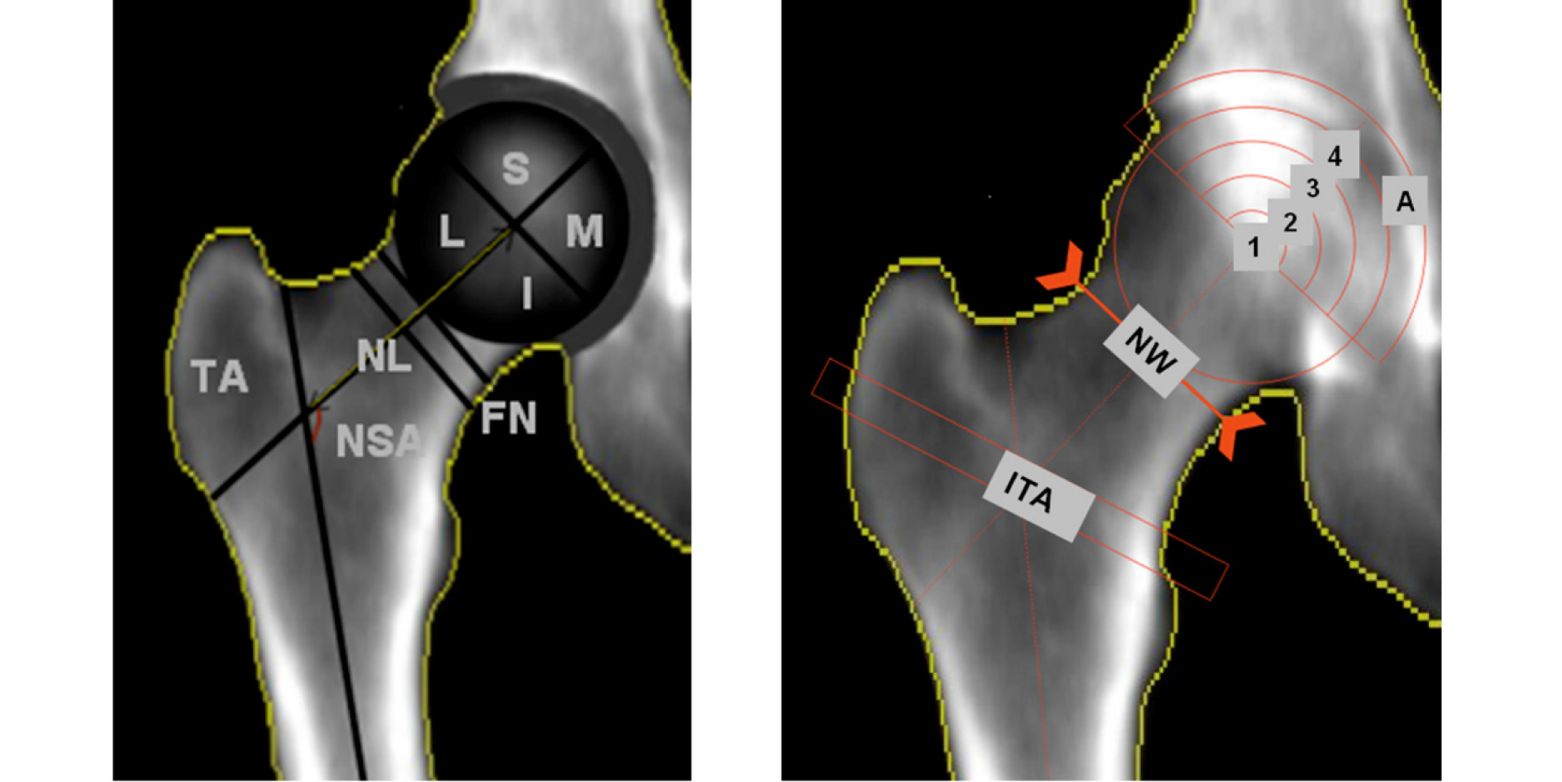
DXA images that show the parameters that are determined in the software for the DXA scan analysis. **a) **Trochanteric area (TA), Neck shaft angle (NSA), femoral neck length (NL): line from the center of the femoral head to the intersection point of the femoral shaft and femoral neck (FN). The femoral head was divided in four quarters: Superior (S), Medial (M), Inferior (I), and lateral (L). **b) **Arcs dividing the upper part of the femoral head in four sub regions ranging from the center of the subchondral region and acetabular area (A), neck width (NW) measured on the narrowest neck region and intertrochanteric area (ITA). For all areas the BMD, BMC and area size were determined.

**Figure 2:**
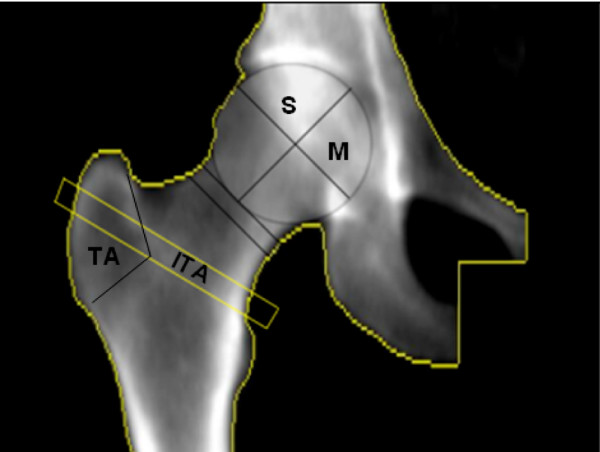
DXA image that shows the parameters of the DXA scan that are part of Model 5, which provides the overall best prediction of OA progression. Superior area size (S), superior and medial (M) BMD and BMC from the femoral head, Intertrochanteric and trochanteric area size (ITA and TA, respectively).

DXA-scans (DPX-Lunar, GE Healthcare, Waukesha, WI, USA) from both hips were made at baseline ensuring 15° internal rotation of the hips, similar to the protocol used for the radiographs. A software tool was developed that enables evaluating bone geometry and density parameters from DXA scans in specified (non-conventional) regions of interest in the hip. Regions of interest (ROI) of which we calculated BMD, BMC and area size included the femoral head (divided in quarter and arcs), femoral neck, acetabulum, trochanteric and inter-trochanteric areas. Figure 1 presents a detailed definition of all the DXA parameters. The analysis was performed using Matlab (version 7.1.0, MathWorks Inc, Natick, Massachusetts, USA). The software calculated the parameters in a semi-automatic way. The major and minor trochanters were indicated manually, as was the size and position of the femoral head according to the location of the bony margins of the acetabulum or acetabular rim, which were used as points of reference; all other parameters were measured automatically. The neck axis was positioned in the middle of the femoral neck, bisecting the centre of the neck. The femoral axis was determined as a line parallel to the femoral shaft passing through the middle point localized between the most external margins of the femur. Geometry parameters and regions of interest (ROI) for BMD, BMC or area measurements included the femoral head, femoral neck, acetabulum, trochanteric and inter-trochanteric areas. Figure 1 and Figure 2 show a detailed definition of all the DXA parameters.

### Progression of hip osteoarthritis

We defined progressive cases as those patients that presented joint space narrowing (JSN); a decreased joint space width (JSW) compared to baseline of twenty percent (20%) or more was considered positive for progression of hip OA according to previously described criteria [20]. It takes into account the big variability in the joint space that exists between individuals. We included in the progression group also those patients that received a total hip replacement (THR) during the two-year follow up.

### Statistical models

We evaluated a number of statistical regression models with different combinations of the following variables: baseline Kellgren and Lawrence (K-L); baseline DXA-based parameters (both geometry and BMD or BMC related parameters); the K-L score difference between the most affected and contralateral hip at baseline (ΔK-L) and the difference between the most affected vs. contralateral DXA-based parameters at baseline (ΔDXA). All models were adjusted for age, weight, height, and sex. To reduce the number of DXA variables to a significant subset we used a backward stepwise method using the Likelihood ratio test. Progression of OA was predicted using five different models: the first Model (1) was used to investigate the contribution of the K-L score of the most affected side to the prediction of progression (K-L); Model 2 was used to investigate the contribution of the DXA based parameters of the most affected side to progression (DXA); Model 3 revealed how the combination of DXA parameters and the K-L score of the most affected side contribute to the prediction of progression (DXA + K-L); Model 4 was used to test if adding the K-L difference within hips to the K-L score of the affected side only improved the prediction of progression of model 1 (K-L + ΔK-L); and Model 5 was used to test if the difference of the most affected (OA) and contralateral hip between the DXA parameters added to the prediction based on K-L score of the affected side (K-L + ΔDXA).

The likelihood-ratio test was used to determine if the differences between the models were significant [21]. Using the software package *R *we calculated *An Information Criterion *(AIC) values of the various models. R is a programming language and open source software environment for statistical computing and graphics widely used for data analysis. AIC is an index of the amount of information that is lost when the model is used to describe the data [22]. The preferred model is the one with the AIC value closest to zero. In all regression models areas under the Receiver Operator Characteristic curves (ROC) were determined and used to compare the discriminatory capacity of the models. The Areas under the Curve (AUC) represent the prediction probability that a randomly selected pair of diseased and non-diseased subjects will be correctly classified. A perfect predictive model has the value AUC = 1.0. Conversely, a non-informative test has AUC = 0.5. True positive and true negative rate were separately analyzed to identify the percentage of OA cases and non-cases correctly predicted by the models. In addition, Nagelkerke R^2 ^was used to measure the proportion of variability in a data set that is accounted for by the statistical models. Nagelkerke's R^2 ^is a modification of the Cox and Snell coefficient to assure that it can vary from 0 to 1. Ten-fold cross validation was used to reduce the error due to overfitting for the statistical estimates (AIC and AUC). All statistical analysis were performed using SPSS, version 14 (SPSS inc., Chicago, Illinois, USA) and R version 2.7.2 (Free Software Foundation Inc., Boston, Massachusetts, USA).

## Results

### Participant characteristics and progressor characteristics

Out of the 222 patients that were enrolled in the trial, 189 patients had DXA scans of sufficient quality to be included in the current study. Using our definition for progression 43 out of 189 patients (22.8%) were considered to have developed radiographic progression of hip osteoarthritis after two years of follow-up (Table 1). Of the 43 patients that progressed, 17 (39.5%) received a total hip replacement and 26 had a JSN of 20% or more. We did not find significant differences in age, sex, weight and height between the progression and non-progression groups (Table 1). The majority of the progressors were found among patients with a K-L score of 2 and 3. There were no progressors in the group with a baseline K-L score of zero (Table 1). JSW decreased with increasing K-L score, with slightly (but not significantly) lower baseline values for the progressor group (Table 2). The biggest differences in BMD or BMC between progressors and non-progressors were found in the regions close to the joint space (superior and medial part of the head and the outer arcs 3 and 4, Table 3 and Figure 1). As expected, these values were higher (Z-score 0.39 to 0.48) for the progressors. The area of the entire femoral head (all four quarters) and the femoral neck width also were significantly higher in the progressor group (Table 3).

**Table 1:** Baseline population characteristics of studied population

Characteristic	n = 189	Progressor n = 43	Non-progressor n = 146
Age (years) mean +/- SD	63.5 +/- 9.0	64.2 +/- 8.7	63.2 +/- 9
- Age 41-60, n (%)	72 (38)	16 (37)	56 (38)
- Age 60-70, n (%)	117 (62)	27 (63)	90 (62)
Female, n (%)	131 (69)	26 (60)	105 (72)
Height, mean +/- SD	1.69 +/- .08	1.69 +/- .08	1.69 +/- .08
Weight, mean +/- SD	78.8 +/- 12.5	80 +/- 11.5	78.5 +/- 12.8
BMI (kg/m^2^), mean +/- SD	27.7 +/- 4.0	27.9 +/- 3.3	27.7 +/- 4.2
K-L score 0	12	0	12
K-L score 1	95	6	89
K-L score 2	57	21	36
K-L score 3	25	16	9

K-L score = Kellgren and Lawrence.

**Table 2:** JSW at baseline and follow up in progressor and non-progressor groups according to KL score at baseline

K-L score	Progressors		Non-progressor	
	JSW bas	JSW fu	JSW bas	JSW fu
**0**	N/A	N/A	3.0 (0.7)	3.0 (0.6)
**1**	2.67 (0.9)	2.31 (1.2)	2.8 (0.5)	2.8 (0.6)
**2**	1.62 (0.83)	1.15 (0.64)	1.89 (0.75)	1.93 (0.73)
**3**	0.75 (0.7)	0.57 (0.7)	0.8 (0.9)	0.8 (1.1)

Values represent JSW in mm (mean and SD) at baseline and two years follow up.

JSW = joint space width; KL score = Kellgren and Lawrence score

**Table 3:** DXA variables for progressors and non-progressors

Variables	Z-score non-progressors	Z-score progressors	Adjusted p-value
***BMC***			
Femoral Neck (FN)	-0.07	0.16	0.17
Intertrochanteric Area (ITA)	0.02	0.07	0.9
Trochanteric area (TA)	-0.01	0.02	0.6
Superior quart femoral head (S)	-0.13	0.44	0.009
medial quart femoral head (M)	-0.10	0.39	0.019
inferior quart femoral head (I)	-0.07	0.24	0.08
lateral quart femoral head (L)	-0.08	0.27	0.06
acetabular arc (A)	-0.10	0.36	0.01
arc4	-0.12	0.45	0.003
arc3	-0.13	0.48	0.001
arc2	-0.11	0.37	0.02
arc1	-0.07	0.24	0.19
***Areas/size***			
Femoral Neck (FN)	-0.08	0.21	0.6
Intertrochanteric area (ITA)	0.02	-0.02	0.16
Trochanteric area (TA)	0	0	0.4
Superior quart femoral head (S)	-0.15	0.47	0.002
medial quart femoral head (M)	-0.12	0.50	0.002
inferior quart femoral head (I)	-0.15	0.47	0.003
lateral quart femoral head (L)	-0.15	0.49	0.003
Acetabular arc (A)	-0.08	0.20	0.04
arc4	-0.01	0.04	0.005
arc3	-0.15	0.06	0.001
arc2	-0.10	0.10	0.007
Arc1	-0.07	0.32	0.2
***Geometry***			
Neck width (NW)	-0.14	0.38	0.04
Neck length (NL)	0.00	-0.04	0.41
Neck shaft angle (NSA)	-0.02	0.08	0.7

Values represent the distance between the mean value of each variable for progressors and non progressors and the population mean in units of the standard deviations. Z is negative when the group's mean is below the population mean. P value was adjusted by gender, age, height and weight.

BMC = bone mineral content

### Model results

The Kellgren and Lawrence score (K-L) proved to be a significant predictor for progression. After cross-validation the area under the Receiver Operator Curve (AUC) for Model 1 was 0.76 (Table 4). The true positive rate (TPR) of this model is 37.2%.

**Table 4:** Models using clinical, radiological and DXA variables

	Variables	% Diff. & p	-2 Log	R^2^	AIC	AUC	TNR	TPR
**1**	KL score affected side:	30.7% (***)	159.5	0.31	163.5	0.76	93.2	37.2
**2**	DXA affected side:		184.2	0.15	192.2	0.69	97.3	9.3
	- BMC medial part femoral head	13.9% (***)						
	- BMC inferior part femoral head	7.2% (***)						
	- BMC femoral neck	5%(*)						
**3**	DXA affected side + KL:		148.6	0.38	158.6	0.83	93.9	34.9
	- BMC medial part femoral head	13.9%(*)						
	- BMC inferior part femoral head	7.2%(*)						
	- BMC femoral neck	5%(**)						
	- KL affected side	NA(***)						
**4**	KL affected side + Delta KL		154.0	0.35	160	0.82	93.9	34.9
	- KL score affected side	NA(***)						
	- Delta KL	32%(*)						
**5**	DXA ROI'S difference:		135.6	0.45	153.6	0.84	91.7	51.2
	- Difference superior area fem head	16.5% (*)						
	- Difference trochanteric area size	2% (*)						
	- Difference BMD sup. part fem. head	5.7% (**)						
	- Difference BMC sup. part fem. head	9% (**)						
	- Difference BMD med. part fem. head	4.6% (**)						
	- Difference BMC med. part fem. head	4% (**)						
	- Difference Intertrochanteric area size	-4.5% (*)						
	- KL score affected side	NA(***)						

The difference in values between affected hip and contralateral side is expressed in percentage (%). Positive values represent an increase in the affected hip. No applicable (NA) in the cases that the variable only reflect the affected side. Level of significance codes: '***' *P *value < 0.001, '**' *P *value < 0.01, '*' *P *value < 0.05. All models were corrected for patient characteristics. TPR and TNR columns correspond to the percentage correctly predicted by the models. *Area under the curve value obtained after 10-fold cross validation process.

AIC = *An Information Criterion*; AUC = areas under the curve; DXA = dual energy X-ray absorptiometry; KL = Kellgren and Lawrence score of the affected side; ROI = rate opf interest; TNR = true negative rate TPR = true positive rate

In the next model we analyzed the DXA scan parameters of the affected side. The backward stepwise regression left only three variables in the model: the BMC of the medial part of the femoral head, the BMC of the inferior part of the femoral head and the BMC of the femoral neck (Model 2). After cross-validation the model's performance was inferior to K-L in Model 1 (AUC = 0.69, Table 4). Similarly the true positive rate (TPR) of this model was lower (9.3%).

In Model 3 we combined the predictors from Model 1 (K-L score) with the predictors from Model 2 (the three BMC DXA variables), which resulted in a model with reasonable good predictive performance after cross-validation (AUC = 0.83). The difference in AUC score of this model with the previous two models proved to be significant (*P *< 0.05). The TPR of 34.92 was slightly less than Model 1, Table 4.

In Model 4 we added the K-L score difference (ΔK-L) between the hips of each patient as a predictor to model 1 (K-L score of the affected side only). Adding ΔK-L resulted in a significant increase in AUC (*P *< 0.05) compared to Model 1. Both the AUC (0.82) and the TPR (34.9%) were similar to the values for Model 3, Table 4.

In the last model (Model 5) we combined K-L of the affected side (Model 1) with the difference in DXA values between the most affected and contralateral hip. The backward regression resulted in a different set of DXA parameters than those identified by Model 2: The area size of the superior part of the femoral head, the area of the major trochanter, the intertrochanteric area and both the BMD and BMC of the superior part and medial part of the femoral head were selected (Figure 2). This model is significantly different to the model that only includes K-L score of the affected side (Model 1) and to the model that uses the K-L score difference and the value of the K-L score of the affected side (Model 4) based on comparing AUC differences after cross-validation (*P *< 0.05). The AUC of Model 5 (0.84) was not different from the AUC of Model 3 (K-L + DXA most affected side; AUC: 0.83), but the model is much better in the prediction of progressive cases (with a TPR of 51.2%). Additionally, this model has the lowest -2Log Likelihood ratio and AIC value (Table 4).

## Discussion

In this study we analyzed how well selected DXA parameters of the hip that were specifically chosen to be relevant for osteoarthritis, together with the accepted Kellgren & Lawrence score contribute to the prediction of OA progression.

We found that both the K-L score and the selected DXA parameters alone were not good predictors for OA progression, with K-L performing marginally better than the DXA parameters alone. Interestingly, when both models were combined the resulting model exhibited a small but significant increase in performance as shown by the increase in AUC. Apparently, the DXA parameters that were investigated in this study refer to measures of OA that are relatively independent of the Kellgren & Lawrence score. Many of the DXA parameters themselves however, were not independent but highly correlated among each other. The number of DXA variables used in the regression models was reduced using the backward stepwise method in the likelihood ratio test. Therefore the resulting regression models are dependent on the backward stepping procedure and other models that include other parameters (representing similar aspects) might work just as well. What is important here is not so much the meaning of the specific parameters used in the regression models, but the potential of DXA parameters for the prediction of OA progression, which justifies a more in depth study.

We further investigated if the prediction based on DXA parameters would improve when the difference between most affected and contralateral side was used rather than the affected side itself. We assumed that looking at the DXA difference between the most affected and contralateral side would correct at least partly for the biological variation in bone sizes and bone density. Thus, this measure could highlight how the disease process has affected the bone and therefore be a better predictor for disease progression. Even though the AUC for the model that included this ΔDXA (Model 5) was only slightly higher than the AUC of Model 3 (DXA parameters of the most affected side and K-L score of the most affected side), the percentage of correctly classified progression cases (TPR) is much higher than in Model 3. Additionally, this model (Model 5), showed a better statistical performance, lowest -2Log, AIC and higher R^2 ^(Table 4: -2Log: 135.6, AIC: 153.6 and R^2^: 0.45) than any other model.

The definition of progression in this study included patients with both JSN (more than 20%) and patients that received a total hip replacement (THR) within the follow-up period of two years [20]. The latter is maybe a possible limitation of this study, because we cannot determine if the THR patients truly exhibited joint space narrowing. We tested the effect of excluding the THR patients to the models in a sensitivity analysis. In all models the exclusion of THR cases affects the percentage of correct predictions and AUC. However, the general trends were similar and the model that included the difference between the most affected- and the contralateral side (Model 5) still remained the best predictive model.

Other limitations of this study are related to the relatively short follow-up and the inaccuracies inherent to the DXA measurements. The limitations of the DXA method itself have been exposed previously by other authors [23]. Radiological progression of osteoarthritis is better defined when patients have longer follow up.

In addition the study population is rather heterogeneous with patients that varied in (subjective) pain scores and ranged from mild OA (K-L 0 and 1) to advanced stages (K-L 2 and 3). It seems likely that the more degenerated joints at baseline progress differently than a joint in the early phase of the disease. In terms of our definition of progression it is clear that advanced OA joints with an already small JSW don't have to progress much to reach a 20% narrowing. The majority of the progressors are in the K-L scores 2 and 3 and since a K-L score of 4 was an exclusion criterion we have no patients with extreme low JSW (Table 2).

Different hypotheses exist about the role of BMD changes during the osteoarthritis process. We had defined different regions of interest of which some were close to the joint with a putative effect on osteoarthritis development. Not only femoral head regions were found to be relevant, but also the more distant regions such as the femoral neck and trochanteric regions. The difference in intertrochanteric area size (between affected and contralateral hip) had a negative correlation with progression and might be the consequence of muscular dysfunction of the hip abductor group that has been found in patients with hip osteoarthritis [24,25].

We also identified an increase in size at the femoral head and trochanter and increased BMD and BMC of the superior and medial part of the most affected femoral head compared to the contra lateral side in the group of patients where the disease progressed (Figure 2). The BMD and BMC increase in the head regions is in concordance with published literature and we suppose that the differences are acquired as part of the osteoarthritis process and subsequent bone adaptation. However we cannot exclude the possibility that some of these left-right differences existed previous to the onset of the disease.

## Conclusions

We have shown that DXA scans of the hip contain information that can be used to predict OA progression. Patients that presented OA progression had a higher BMC in the medial and inferior region of the femoral head compared to those that did not progress. Also, the bone mass in these regions was higher in the most affected hip compared to the contralateral side. These differences between the most affected hip and the contralateral hip appear promising to predict progression of the disease. Further study of DXA scans with improved resolution could lead to the development of useful clinical tools to diagnose OA and predict the chances of fast progression.

## Abbreviations

AIC: *an information criterion*, it is a measure of the goodness of fit of an estimated statistical model. It is a tool for model selection; AUC: area under the curve; BMC: bone mineral content; BMD: bone mineral density; BMI: body mass index; DXA: dual energy X-ray absorptiometry; FPR: false positive rate; JSN: joint space narrowing; JSW: joint space width; K-L: Kellgren and Lawrence Score; OA: osteoarthritis; ROI: region of interest; ROC: Receiver Operator Characteristic curves; TPR: true positive rate; ΔDXA: difference in DXA measurements within the hips of each subject; ΔK-L: difference in KL score within the hips of each subject.

## Competing interests

The authors declare that they have no competing interests.

## Authors' contributions

MC participated in the design of the study, analysis of DXA images, performed the statistical analysis and drafts the manuscript. JL conceived the study and elaboration of the program used to analyse the DXA images. RF participated in the statistical analysis and helped to draft the manuscript. RR selected the cohort and collected patient's information, SB participated in the study design and helped to draft the manuscript. HW conceived the study, participated in its design and coordination, and helped to draft the manuscript. JW participated in the design of the study and design of the program used to analyse the DXA images, helped to perform the statistical analysis and draft the manuscript. All authors read and approved the final manuscript.
